# The impact of text message reminders on cryotherapy uptake among women testing positive for HPV in western Kenya: a prospective cohort study

**DOI:** 10.1186/s12905-023-02842-x

**Published:** 2024-01-13

**Authors:** Yujung Choi, Saduma Ibrahim, Lawrence P. Park, Elizabeth A. Bukusi, Megan J. Huchko

**Affiliations:** 1 Department of Population Health Sciences, Duke University School of Medicine, Durham, NC USA; 2 Kenya Medical Research Institute, Nairobi, Kenya; 3 Duke Global Health Institute, Duke University, Durham, North Carolina, USA; 4 Department of Obstetrics and Gynecology, Duke University, Durham, North Carolina, USA

**Keywords:** Cervical cancer, HPV, Screening, Text messaging, mHealth, Kenya

## Abstract

**Background:**

Mobile health (mHealth) has become an increasingly popular strategy to improve healthcare delivery and health outcomes. Communicating results and health education via text may facilitate program planning and promote better engagement in care for women undergoing human papillomavirus (HPV) screening. We sought to develop and evaluate an mHealth strategy with enhanced text messaging to improve follow-up throughout the cervical cancer screening cascade.

**Methods:**

Women aged 25–65 participated in HPV testing in six community health campaigns (CHCs) in western Kenya as part of a single arm of a cluster-randomized trial. Women received their HPV results via text message, phone call, or home visit. Those who opted for text in the first four communities received “standard” texts. After completing the fourth CHC, we conducted two semi-structured focus group discussions with women to develop an “enhanced” text strategy, including modifying the content, number, and timing of texts, for the subsequent two communities. We compared the overall receipt of results and follow-up for treatment evaluation among women in standard and enhanced text groups.

**Results:**

Among 2368 women who were screened in the first four communities, 566 (23.9%) received results via text, 1170 (49.4%) via phone call, and 632 (26.7%) via home visit. In the communities where enhanced text notification was offered, 264 of the 935 screened women (28.2%) opted for text, 474 (51.2%) opted for phone call, and 192 (20.5%) for home visit. Among 555 women (16.8%) who tested HPV-positive, 257 (46.3%) accessed treatment, with no difference in treatment uptake between the standard text group (48/90, 53.3%) and the enhanced text group (22/41, 53.7%). More women in the enhanced text group had prior cervical cancer screening (25.8% vs. 18.4%; *p* < 0.05) and reported living with HIV (32.6% vs. 20.2%; *p* < 0.001) than those in the standard text group.

**Conclusions:**

Modifying the content and number of texts as an enhanced text messaging strategy was not sufficient to increase follow-up in an HPV-based cervical cancer screening program in western Kenya. A one-size approach to mHealth delivery does not meet the needs of all women in this region. More comprehensive programs are needed to improve linkage to care to further reduce structural and logistical barriers to cervical cancer treatment.

## Introduction

Although cervical cancer is largely preventable through effective screening and vaccination for human papillomavirus (HPV), it still remains a major public health burden in much of the world [1]. Approximately 90% of cervical cancer incidence and mortality occur in resource-limited settings where the lack of prevention is compounded by limited treatment options [2]. In much of East Africa, including Kenya, cervical cancer is the most frequent cause of cancer-related death among women [3–5]. While Kenya has adopted the World Health Organization (WHO) recommendations for simplified HPV-based screening strategies, there are major gaps in implementation and substantial loss-to-follow-up after screening [6, 7]. Reasons for loss-to-follow-up include transportation costs and distance to treatment facilities, stigma, lack of social support, and low levels of personal risk perception or knowledge about HPV and cervical cancer [8, 9].

Offering self-collected HPV-testing in the community in Western Kenya has been shown to be an effective screening strategy. However, while community-based screening can substantially improve screening rates over baseline, and is more cost-effective than facility-based testing [10, 11], a crucial limitation is the loss to follow-up of women who tested positive for HPV. Novel strategies to increase attendance at both screening and follow-up include visit navigators, transportation vouchers, treatment incentives, and health messaging via mobile phones (mHealth). Several programs have evaluated the combination of reminder telephone calls and travel incentives, which were shown to improve follow-up [12, 13]. However, this combination of interventions is labor intensive, costly, and places additional burdens on health facility staff. mHealth strategies utilizing text messages have the potential to reach large numbers of people through automated messaging about health conditions and services, while requiring relatively low costs and administrative burdens [14]. One possible solution would be to use text messages as a way to delivering cervical cancer screening results, health messaging and logistical information about follow-up [15].

mHealth solutions may be particularly suited to Kenya, where 78% of households own or have access to mobile phones [16]—more than those who have access to public water and sanitation services—and many use their mobile phones frequently, as evidenced by over $108 million in cash transfers carried out through mobile phones daily. Mobile phones have been shown to be effective in educating patients about sensitive health-related issues that require confidentiality in various health domains in Kenya, such as HIV prevention [17, 18], family planning [19], and sexually transmitted infections [20]. Text messages, in particular, have been found useful for reminding patients about medication adherence [20, 21] and increasing preventive health visits and outpatient clinic attendance in many low- and middle-income countries (LMICs) [22, 23].

As part of a two-phase trial of implementation strategies for cervical cancer screening in western Kenya, our team introduced text messaging to deliver HPV test results and follow-up plans to women [24, 25]. In the first phase, while text messaging was a popular and efficient method of results delivery, it did not result in higher rates of treatment uptake when compared with notification through phone calls or home visits. From individual interviews at the time of treatment, we found that women wanted more clear and personalized information when receiving their results. Therefore, in the second phase, we sought to develop and evaluate an intensified mHealth strategy with enhanced text-messaging to improve rates of follow-up with treatment after a positive HPV test through improved understanding of HPV, treatment logistics and information to share with their partners. This paper describes the modifications made to the content of text messages informed by feedback from focus group discussions. It further examines the acceptability of enhanced text messages and their impact on treatment uptake by comparing two different community arms in the second phase: one employing standard text messaging, and the other utilizing enhanced text messaging.

## Methods

This study was part of a two-phase cluster-randomized trial evaluating community-based cervical cancer prevention strategies using HPV self-sampling in Migori County, Kenya (ClinicalTrials.gov identifier: NCT02124252–28/04/2014). The two-phase design allowed the study team, including community partners, to collect feedback and evaluate uptake data to iteratively improve the implementation strategy, with a focus on improving follow-up, between phases. Results from the first phase showed that the community-based HPV testing model had higher uptake and lower program costs compared to screening in health facilities [10, 11, 26]. This paper presents a mixed-method sub-study within the single-arm, second phase of the randomized trial, exploring the development, acceptability, and impact of an enhanced mHealth strategy on cryotherapy uptake among women who test positive for HPV. In the second phase, we offered the more effective community-based screening coupled with optimized linkage to treatment strategies in six communities. The enhanced linkage strategy included decentralization of treatment sites, increasing from one to four; increased provider training and supervision, and texts tailored to provide further education on cervical cancer and reminders for treatment. The study activities described below were nested within the second phase.

### Participants and setting

Participants included women between the ages of 25 and 65 years, with an intact uterus and cervix, who resided within the six study communities in Migori County, Kenya. Study communities were defined by the sub-locations assigned to one government health facility, with an overall population of approximately 5000. To avoid spillover, we identified communities with non-adjacent borders that had not participated in the first phase of the study. Prior to carrying out the community heath campaigns (CHCs), the study team conducted door-to-door enumeration to characterize the study communities more accurately, which is presented in detail elsewhere [26].

### Structure of community health campaign with standard text messaging

The process of education and self-collection of specimens for HPV testing at the CHCs is described in detail elsewhere [26]. After collection, women provided their preference for receiving HPV test results (text message, phone call, and home visit). We used the *care*HPV test (QIAGEN, Germantown, MD) to collect and process samples, with a goal of providing results to participants within 2 weeks. Text notification provided through the Frontline SMS™ program (https://www.frontlinesms.com/). Receipt of HPV test results via text was considered successful if the program confirmed the transmission of text message, meaning the participant’s phone was on and the SIM card was valid or phone line was active [24].

The text-based HPV test results notification took into consideration participants’ HIV status and HPV test results: 1) HPV negative and HIV positive; 2) HPV negative and HIV negative; 3) HPV positive; and 4) inconclusive HPV test result (Table 1). Based on their test result, participants also received guidance regarding their next cervical cancer screening and any necessary treatment following a positive HPV test.

**Table 1:** Examples of the standard and enhanced texts

Standard text	Enhanced text
**Post-screening text** N/A	**Post-screening text** Thank you for screening, you will receive results soon! Remember, HPV causes cervical cancer, but HPV doesn’t mean you have cancer. Free treatment is available.
**Treatment** HPV−/HIV+ client Hallo (recipient name). Thank you for taking cervical cancer screen test! Your result was Negative! Visit your nearest clinic after 1 year (2019) for another test. Please call or flash (Study Phone) if you have questions. HPV−/HIV- client Hallo (recipient name). Thank you for taking cervical cancer screen test! Your result was Negative! Visit your nearest clinic after five years (2023) for another test. Please call or flash (Study Phone) if you have questions. HPV+ client Hallo (recipient name). Thank you for taking cervical cancer screen test! Your results showed that you have HPV. Please come to (facility) to talk about treatment options. Treatment will be available until (Date). Call or flash (Study Phone) if you have questions. Indeterminate HPV test Hallo (recipient name). Thank you for taking cervical cancer screen test! Sorry we were not able to evaluate your sample. We would like to collect another sample from you. Please call or flash (Study Phone) to discuss plan for repeat testing.	**Treatment (same as previous)** HPV−/HIV+ client Hallo (recipient name). Thank you for taking cervical cancer screen test! Your result was Negative! Visit your nearest clinic after 1 year (2019) for another test. Please call or flash (Study Phone) if you have questions. HPV−/HIV- client Hallo (recipient name). Thank you for taking cervical cancer screen test! Your result was Negative! Visit your nearest clinic after five years (2023) for another test. Please call or flash (Study Phone) if you have questions. HPV+ client Hallo (recipient name). Thank you for taking cervical cancer screen test! Your results showed that you have HPV. Please come to (facility) to talk about treatment options. Treatment will be available until (Date). Call or flash (Study Phone) if you have questions. Indeterminate HPV test Hallo (recipient name). Thank you for taking cervical cancer screen test! Sorry we were not able to evaluate your sample. We would like to collect another sample from you. Please call or flash (Study Phone) to discuss plan for repeat testing.
**Follow-up text** N/A	**Treatment Reminders** Your treatment for HPV will be on XXX at (facility). Come between XX and XX. We encourage you to bring your partner or relative. (Study Phone) for questions.
**Post-treatment text** N/A	**Post-treatment text (One day after treatment)** The treatment you had is important to your health. To allow healing and prevent re-infection, please avoid intercourse for 6 weeks. (Study Phone) for questions.

Women who opted to receive their HPV test result notification via text in the standard group received one text message. For those who did not follow-up for treatment, second and third attempts for results notification for women were completed by phone call or home visit. Phone call and home visit strategies were deemed successful if the participant was reached and was given their results directly by study staff. Our study staff attempted up to four phone calls or three home visits before determining that a participant was unable to be reached.

### Development of the enhanced text messaging strategy

After the fourth CHC, in July 2018, we conducted two semi-structured focus group discussions (FGDs) with female participants who had been previously screened during the first phase of the trial and had opted for a cell phone-based strategy (text of phone call) for their results notification. These participants were identified and recruited by community health volunteers for their ability to actively engage and provide valuable feedback. Each FGD consisted of 10 participants and lasted for 2.5 hours. We explored myths and misperceptions related to HPV found in qualitative data from the first phase, such as misunderstanding of how HPV is treated, what causes HPV, and meaning of a positive result. We also examined women’s preferences for sharing information with their partners, barriers to accessing treatment, and the rationale behind their choice of text or phone call for receiving HPV test results. FGD participants were asked to help identify appropriate content and wording to develop messages that would most resonate with women.

**Fig. 1:**
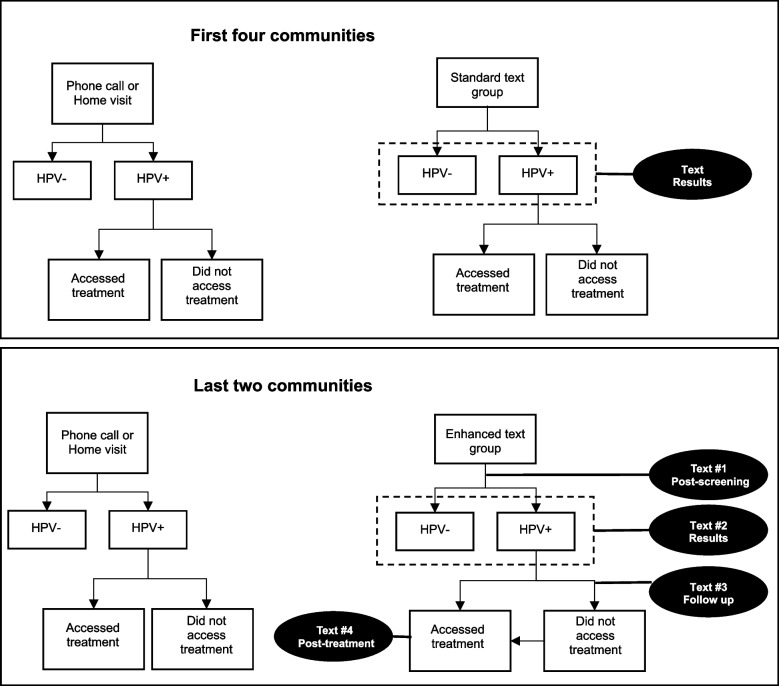
Flowchart of the results notification strategies

Given the straightforward nature of participants’ responses to our research topics, we employed structural coding to synthesize our findings. In response to the FGD feedback, the enhanced text messaging strategy included changes in the timing, number, and content of messages (Fig. 1). Messages were developed to be more clear, concise, and specific to the patient (Table 1). To ensure understanding, women who opted to receive their results via texts were shown examples of texts at the time of HPV screening. Messages were sent out more frequently; in addition to a text with their results, women received a brief message thanking them for screening and additional treatment reminders if they tested positive. Treatment reminder text messages were tailored to address common barriers in accessing treatment, which may include providing location of clinics, time of appointment, and a possible description of transport options.

### Evaluation of the enhanced text messaging strategy

The enhanced text messaging strategy was deployed in the last two study communities. We collected information about participants through a structured questionnaire administered at three time points: at the CHCs prior to HPV testing (pre-test), immediately after HPV testing (post-test), and after treatment for those who screened positive (follow-up). Our primary outcomes for this study were receipt of HPV test results and treatment uptake. Prior to screening, we collected information of sociodemographic characteristic, clinical information, and behavior regarding their phone use, frequency, and barriers to phone ownership, access, or use, if any. At the follow up, we asked participants about their acceptability of text messaging and the role of receiving text messages in their decision to access treatment.

### Treatment

Women who tested HPV positive were referred for evaluation for cryotherapy at local health facilities. Treatment was provided by trained clinic providers, and the study staff kept track of participants who successfully received treatment and their date of treatment. Treatment was available for up to 3 months at each health facility after participants who tested HPV positive were notified of their result in their respective community. To calculate time to treatment, we only included women who accessed treatment through April, 2019.

### Statistical analysis

Descriptive statistics were used to compare the baseline characteristics of the women in the first four communities and the last two communities, as well as standard and enhanced text groups. To test bivariate relationships between treatment uptake and categorical demographic characteristic variables, we performed chi-squared tests. We used Kruskal-Wallis test to evaluate continuous variables and the median time and interquartile range in days between screening and notification, notification and treatment access, and screening to treatment access. *P* values of < 0.05 were considered statistically significant. All analyses were performed using STATA version 16 (College Station, TX: StataCorp LP).

## Results

### Focus groups

#### What FGD participants liked and disliked about results notification via text messaging

Participants highlighted several key benefits of receiving their results via text, including convenience, privacy, and control over information sharing. Some women felt there was a lower chance of missing their results when delivered via text. They liked the flexibility of receiving information at any time, even if their phone was off, as it could be accessed later at their convenience, unlike phone calls with a specific place and time. One 26-year-old participant noted, “Even if my phone was off by the time they are sending the message, I will still get the message.” Another 27-year-old participant expressed, “I am not always with my phone all the time. They [study team] can call and at that time I don’t have it, and they may not call again. That means I will miss it, that’s why I prefer texting to a phone call.” Furthermore, some participants appreciated that they could refer back to the text at a later time. “With text, you’ll have that information as long as you want it, that’s why I preferred text to phone call.” (A 27-year-old participant).

Participants also expressed a sense of comfort when receiving sensitive information via text due to the privacy it offered. One 26-year-old stated, “I’ll use my own password to read the text…no other person will have access to it.” Several participants specifically noted the increased privacy with text messages compared to other result notification strategies. A 42-year-old participant mentioned, “I chose texting because it has privacy. You have to shout when making or receiving a phone call that even those whom you don’t want to have your information will have it. With text, you read it on your own.” Another participant shared, “I had the opportunity for them [study team] to do a home visit, but I chose not to because people in our village will talk a lot and make statements.” (A 52-year-old participant).

An important aspect of privacy was control over how they share their information. They valued having information readily accessible, giving them the autonomy to decide when and with whom to share it. A 42-year-old participant explained, “I’ll receive a text on my phone and I’m the one who will read it. If I want to share it, that will be up to me.” Another woman noted, “I will not be able to explain everything well to him (my husband), but with the text, he can read and get full information.” (A 26-year-old participant).

For one participant, the extent of control over sharing information was tied to the specific HPV test result they received. This 50-year-old participant explained, “I preferred text because it allowed me to review the information first before sharing the good news of my negative test result with my husband.” However, with a positive HPV result, a few participants held a contrasting view and preferred not to receive their HPV results via text due to the emotional distress it may cause. One 31-year-old participant mentioned being “stressed to death [if they tested positive for HPV]”, while a 25-year-old participant expressed a desire for privacy as they “didn’t want anybody to know and wanted to avoid stigma [related to HPV].” As a result, they preferred to receive their results through a phone call.

In addition to the fear around receiving a positive HPV diagnosis via text, there were other disadvantages to receiving results and other health information in this manner, including inability to ask follow-up questions, communication barriers, and possible unfamiliarity with technology. Despite being provided with contact information for the clinical team, a primary concern was the lack of immediate access to a knowledgeable provider to answer questions or provide more counseling and information about treatment. A 35-year-old woman who opted for phone calls said, “If I had any questions, I could not get my answers right there,” while a 25-year-old participant shared that “you can be counseled through a phone call which is not possible with text. It [phone call] gives you an opportunity to ask questions.”

Some participants felt that there would be communication barriers over text. One 40-year-old participant stated, “I preferred phone call because I will have to talk to the person in a language that I understand well.” Although women were asked about their language preference, they remained uncertain about whether the message would be easily comprehensible, suggesting underlying concerns about the complexity of the information provided. A 25-year-old participant reported, “I didn’t know the type of language they were going to use in sending that text.” For one participant, communication barriers would potentially be compounded by lack of familiarity with text messaging. This 32-year-old participant explained, “In case I would test positive, I will know when and where to go for treatment. Through a text, I cannot even ask that. I don’t know how to text.”

#### Ideas to improve text messaging reported by FGD participants

Women suggested the messages be simple, short, personalized, and the information conveyed in the messages should be educational to the recipient as well as the recipient’s family. Some women commented that texts should be concise because it would make the readers lose interest and that texts should address participants by their names. They also recommended that the messages be sent frequently. One woman reported that notification should be sent 3–4 days prior to actual treatment and should include the specific date and time of when each woman should visit the clinic for treatment. Based in these results, we developed the strategy described above with the content shown in Table 1.

### Pilot of the enhanced text messaging strategy

Between February and November 2018, 3303 women participated in cervical cancer screening with self-collected HPV tests offered through CHCs in six communities (Table 2). Of the 2368 women who underwent cervical cancer screening in the first four communities, almost half (49.4%) chose to receive HPV test results via phone call and less than one-quarter (23.9%) opted for text, making it the least acceptable notification method. In the last two communities, where enhanced text messaging notification was offered, over half (51.2%) of the 935 screened women opted for phone call, followed by more than one-quarter (28.2%) opting for text. Among all participants, 555 (16.8%) tested HPV positive, and 257 (46.3%) of the HPV-positive women accessed treatment. HPV rates (15.9% vs. 15.5%; *p* = 0.943) and treatment uptake (53.3% vs. 53.7%; *p* = 0.928) did not vary between standard and enhanced text groups.

**Table 2:** Demographic and clinical characteristics of participants who participated in HPV-based cervical cancer screening by notification method and communities

Variable	First four communities *n* = 2368	Last two communities *n* = 935	*p*-value	Standard text group *n* = 566	Enhanced text group *n* = 264	*p*-value
Age, mean (sd)	38.6 (11.5)	37.1 (10.7)	0.004	34.2 (8.7)	33.1 (7.4)	0.238
Marital status			0.551			0.400
Married	1808 (76.4)	704 (75.3)		472 (83.4)	206 (78.8)	
Separated/divorced	31 (1.3)	8 (0.9)		4 (0.7)	2 (0.8)	
Single	35 (1.5)	13 (1.4)		9 (1.6)	7 (2.7)	
Widowed	494 (20.9)	210 (22.5)		81 (14.3)	47 (17.8)	
Education			0.488			0.518
None/Some primary	1475 (62.3)	573 (61.3)		190 (33.6)	101 (38.3)	
Completed primary	536 (22.6)	198 (21.2)		180 (31.8)	81 (30.7)	
Some secondary	166 (7.0)	74 (7.9)		85 (15.0)	30 (11.4)	
Completed secondary	122 (5.2)	57 (6.1)		67 (11.8)	29 (11.0)	
College and beyond	69 (2.9)	33 (3.5)		44 (7.8)	23 (8.7)	
Occupation			< 0.001			< 0.001
Unemployed	250 (10.6)	79 (8.5)		64 (11.3)	21 (8.0)	
Business	884 (37.3)	576 (61.6)		218 (38.5)	155 (58.7)	
Farming	919 (38.8)	202 (21.6)		188 (33.2)	52 (19.7)	
Other	315 (13.3)	78 (8.3)		96 (17.0)	36 (13.6)	
Number of children, mean (sd)	5.0 (2.9)	4.5 (2.5)	0.005	4.2 (2.4)	4.0 (2.3)	0.382
Number of children under 13, mean (sd)	2.0 (1.6)	2.1 (1.6)	0.072	2.3 (1.5)	2.3 (1.5)	0.719
Prior cervical cancer screening, n (%)	305 (12.9)	193 (20.7)	< 0.001	104 (18.4)	68 (25.8)	0.015
Type of cervical cancer screening received			< 0.001			0.023
VIA/VILI	253 (83.2)	131 (67.9)		85 (82.5)	43 (63.2)	
Pap smear	9 (3.0)	4 (2.1)		1 (1.0)	0	
HPV	38 (12.5)	52 (26.9)		15 (14.6)	22 (32.4)	
Don’t know	4 (1.3)	6 (3.1)		2 (1.9)	3 (4.4)	
Prior cervical cancer screening result			0.239			0.187
Positive	5 (1.6)	8 (4.2)		2 (1.9)	3 (4.4)	
Negative	269 (88.2)	167 (86.5)		95 (91.4)	64 (94.1)	
Don’t know	31 (10.2)	18 (9.3)		7 (6.7)	1 (1.5)	
Prior cervical cancer treatment			0.196			0.095
Yes	3 (1.0)	6 (3.1)		0	3 (4.4)	
No	297 (96.7)	185 (95.4)		102 (98.1)	64 (94.1)	
Don’t know	7 (2.3)	3 (1.6)		2 (1.9)	1 (1.47)	
Prior HIV testing	2283 (96.4)	906 (96.9)	0.329	555 (98.1)	259 (98.1)	0.312
HIV Status			< 0.001			< 0.001
Positive	459 (20.3)	315 (34.9)		112 (20.2)	84 (32.6)	
Negative	1806 (79.7)	587 (65.1)		443 (79.8)	174 (67.4)	
Currently enrolled in HIV Care	458 (99.8)	313 (99.4)	0.359	111 (99.1)	83 (98.8)	0.837
Family planning			0.008			0.933
Yes	921 (38.9)	409 (43.7)		300 (53.1)	141 (53.4)	
No	1415 (59.8)	508 (54.3)		265 (46.9)	123 (46.6)	
Not sexually active	29 (1.2)	18 (1.9)		0	0	
Tested positive for HPV through this study	403 (17.0)	152 (16.3)	0.594	90 (15.9)	41 (15.5)	0.943
Completed treatment	182 (45.2)	75 (49.3)	0.378	48 (53.3)	22 (53.7)	0.928

Compared to women in the first four communities, women in the last two communities were younger (37.1 years vs. 38.6 years; *p* = 0.004), had fewer children (4.5 vs. 5; *p* = 0.005), had higher rates of cervical cancer screening prior to the CHCs (20.7% vs. 12.9%; *p* < 0.001), with higher proportion of women having completed HPV testing in the past (26.9% vs. 12.5%; *p* < 0.001), and were more likely to report a positive HIV status (34.9% vs. 20.3%; p < 0.001) and engage in family planning (43.7% vs. 38.9%; *p* = 0.008). The similar differences were also observed between standard and enhanced text groups. More women had undergone cervical cancer screening prior to the CHCs (25.8% vs. 18.4%; *p* < 0.05) and reported living with HIV (32.6% vs. 20.2%; *p* < 0.001) in the enhanced text group than those in the standard text group.

Among all women who attended CHCs, 2749 (83.2%) women reported using cell phones daily (Table 3). More women who opted for texts reported owning their own phone (92.5%) and being comfortable with reading and writing texts and receiving sensitive information via text than those who opted for phone calls or home visit (*p* < 0.001). However, women were less likely to share their positive HPV test result with their partners if they opted for texts compared to those who opted for phone calls and home visit. For those who opted for texts, 12.6% requested their results via phone call or 2.1% home visit in the event of a positive test result.

**Table 3:** Phone usage experience and preference by notification method

Variable	Overall *n* = 3303	Phone call *n* = 1650	Text *n* = 829	Home visit *n* = 824	*p*-value
Use mobile phone	2749 (83.2)	1597 (96.8)	811 (97.8)	341 (41.4)	< 0.001
Own phone					< 0.001
Self	2355 (86.0)	1408 (88.3)	750 (92.5)	197 (58.8)	
Family (spouse, child)	359 (13.1)	175 (11.0)	59 (7.3)	125 (37.3)	
Friends, neighbors	26 (1.0)	11 (0.7)	2 (0.3)	13 (3.9)	
Preferred method of receiving negative test result					< 0.001
Text	859 (26.0)	30 (1.8)	823 (99.3)	6 (0.7)	
Phone call	1638 (49.6)	1620 (98.2)	6 (0.7)	12 (1.5)	
Home visit	806 (24.4)	0	0	806 (97.8)	
Preferred method of receiving positive test result					< 0.001
Text	745 (22.6)	37 (2.2)	708 (85.4)	0	
Phone call	1670 (50.6)	1566 (94.9)	104 (12.6)	0	
Home visit	888 (26.9)	47 (2.9)	17 (2.1)	824 (100)	
Comfort with reading texts					< 0.001
Very comfortable	651 (19.8)	330 (20.1)	277 (33.4)	44 (5.4)	
Comfortable	1455 (44.3)	774 (47.0)	509 (61.4)	172 (21.3)	`
Not comfortable	295 (9.0)	131 (8.0)	19 (2.3)	145 (17.9)	
Not comfortable at all	68 (2.1)	42 (2.6)	2 (0.2)	24 (3.0)	
Cannot read	815 (24.8)	369 (22.4)	22 (2.7)	424 (52.4)	
Comfort with writing texts					< 0.001
Very comfortable	645 (19.7)	323 (20.0)	281 (34.1)	41 (5.1)	
Comfortable	1277 (39.0)	657 (40.0)	475 (57.6)	145 (18.0)	
Not comfortable	372 (11.4)	179 (10.9)	33 (4.0)	160 (19.8)	
Not comfortable at all	68 (2.1)	44 (2.7)	1 (0.1)	23 (2.9)	
Cannot read	913 (27.9)	440 (26.8)	35 (4.2)	438 (54.3)	
Comfort with receiving confidential information via text					< 0.001
Very comfortable	671 (21.1)	331 (20.1)	298 (36.0)	42 (5.7)	
Comfortable	1285 (40.5)	660 (40.9)	483 (58.4)	142 (19.4)	
Not comfortable	893 (28.1)	477 (29.5)	38 (4.6)	378 (51.5)	
Not comfortable at all	327 (10.3)	147 (9.1)	8 (1.0)	172 (23.4)	
Notified on first attempt	2443 (74.0)	899 (54.5)	829 (100)	715 (86.8)	< 0.001

There was a significant difference in notification of results at first attempt across the text, phone call, and home visit categories (*p* < 0.001) (Table 3). All women who opted for text received their test result at first attempt, followed by those who opted for home visit (86.8%) and phone calls (54.5%). For those who opted for text and accessed treatment, most (82.5%; *p* < 0.001) did so after receiving first text notification while significantly fewer women sought treatment after second (10.9%) and third text notifications (6.6%).

The median time it took from screening to notification of test results varied by notification method, with text messaging strategy delivering the results most efficiently (16 days; p < 0.001), followed by home visit (20 days) and phone calls (31 days) (Table 4). HPV positive women who opted for text messaging took the longest time to access treatment after receiving their test results (25 days) while those who opted for phone calls had the shortest (7 days).

**Table 4:** Impact of actual notification strategies on treatment uptake and time to treatment

**Phone calls**	**First four communities *n* = 1168**	**Last two communities *n* = 481**	***p***-**value**
HPV Positive, n (%)	195 (16.7)	84 (17.5)	0.705
Completed treatment n (%)	83 (42.6)	40 (47.6)	0.435
Time duration, median days, (Q1, Q3)			
Screening to Notification (*n* = 1649)	30 (21, 55)	35 (14, 62)	0.087
Notification to Treatment (*n* = 123)	9 (4, 34)	3 (2, 14.5)	0.002
Screening to Treatment (*n* = 123)	34 (27, 67)	29 (13.5, 59)	0.068
**Text messaging**	**First four communities with standard text *n*** **= 567**	**Last two communities with enhanced text *n*** **= 262**	***p*-value**
HPV Positive, n (%)	90 (15.9)	40 (15.3)	0.824
Completed treatment n (%)	48 (53.3)	22 (55.0)	0.860
Time duration, median days, (Q1, Q3)			
Screening to Notification (*n* = 829)	18 (13, 21)	12 (10, 50)	0.015
Notification to Treatment (*n* = 70)	25 (9, 71)	24.5 (10, 55)	0.894
Screening to Treatment (*n* = 70)	52 (22, 96)	61.5 (25, 77)	0.690
**Home visits**	**First four communities *n*** **= 632**	**Last two communities *n*** **= 192**	***p*-value**
HPV Positive, n (%)	118 (18.7)	28 (14.6)	0.194
Completed treatment, n (%)	51 (43.2)	13 (46.4)	0.758
Time duration, median days, (Q1, Q3)			
Screening to Notification (*n* = 824)	20 (15, 30)	17 (12, 46)	0.491
Notification to Treatment (*n* = 64)	11 (6, 26)	6 (3, 14)	0.063
Screening to Treatment (*n* = 64)	34 (26, 67)	58 (17, 63)	0.683

## Discussion

We sought to develop an enhanced text messaging strategy to increase completion of the cervical cancer screening cascade in a community-based HPV screening program in partnership with women in western Kenya. We found that, although providing women various options for notification was valued, the chosen notification modality had no effect on treatment uptake, which remained around 50%. Treatment uptake did not improve after incorporating an in-person review of text content, increased frequency, and enhanced text messaging, which was clearer, more concise, and more personalized.

Besides the enhanced text messaging strategy, our team also aimed to make cervical cancer prevention services more accessible and reduce structural barriers by increasing treatment sites and providing additional training and supervision for medical providers in cervical cancer treatment. Overall, treatment uptake did not differ across notification methods. However, women in the communities where the enhancement measures were implemented accessed cervical cancer treatment sooner than women in other communities after receiving their positive HPV result by phone call or home visit. This decrease in time from HPV result notification to treatment may be explained by the enhanced linkage to care strategies and in-person contact with study staff, rather than via text, to counsel women or address their apprehension toward treatment. Similar to our study, one study in Tanzania found that one-way text messages had no effect on the follow-up screening rate among HPV-positive women and instead suggested that provider-initiated phone calls to educate women on the importance of rescreening may be more effective [27].

While we did not observe a greater treatment uptake with the text messaging strategy compared to phone calls or home visits—in fact, time before accessing treatment was longest in the text messaging group—it is important to highlight that the text messaging strategy was also not associated with a lower treatment uptake. The delay in accessing treatment among women who received enhanced text messages and tested positive for HPV in our study differed from a study in Argentina, where text messages served as a cue to action for women to visit the health center to obtain their HPV test results [28]. In their study, 69% visited a health center within 7 days of receiving the text, including 7.5% on the same day. Notably, their study area primarily consisted of urban populations, whereas our study focused on rural communities in Kenya. The limited impact of the text enhancements in our study suggests that there are higher structural barriers to treatment acquisition in this setting that are difficult to offset by enhanced text messaging strategy alone. Such barriers include a long travel distance to the clinic, transportation costs, and a misalignment between work and clinic hours. Similar to our study, a pilot study of community-based HPV self-sampling in rural Uganda, which used SMS for result notifications, found that only 22% of women with positive HPV results attended the clinic for follow-up, identifying transportation challenges as a significant barrier [29]. However, one study based in Tanzania showed that women who received a transportation voucher via text to return to the clinic for cervical cancer screening, as well as 15 texts promoting behavioral change, were 1.53 times more likely to attend screening than those who only received the texts [30]. Although the overall screening uptake was relatively low in the study, their findings highlight the potential impact of mHealth in reducing socioeconomic and systemic barriers for women to access cervical cancer services, especially in rural areas.

Most women felt comfortable receiving either test result via text. However, it is notable that some women chose to receive results via text in the case of negative HPV test results, but via phone calls or home visits if the results were positive. These findings are critical for understanding the gaps in the cervical cancer care continuum. One study in South Africa suggested that in case of abnormal Pap smear results, a text should instruct the women to come to the clinic where the results are then shared during face-to-face discussions with a medical provider [12], given the concerns around privacy of texts and fear of stigma—an important consideration when women may not have their own phone and may share it with their family. Another study based in Argentina used a text messaging strategy to connect women with triage Pap post-HPV testing and to inform women about their HPV test result availability while replacing the term “HPV-testing” with the term “self-collection.” The authors hypothesized that this is one of the ways that helped women reduce concerns related to privacy and increased clinic attendance rates in their study [28, 31]. Although our study team informed women of their positive HPV test result via text and used the term “HPV,” we attempted to reduce stigma toward HPV and ensure confidentiality by asking women to choose their preferred results notification method (phone call, text, or home visit) depending on their HPV test result (positive or negative), making this process as individualized as possible. Nonetheless, more research should be conducted to develop a culturally tailored text intervention for improving treatment uptake.

The challenges inherent in text messaging highlight the advantages of and potential need for greater individual interaction via phone calls or home visits to provide education and link women to treatment. In fact, in our FGDs, women reported that the inability to ask follow-up questions was a negative aspect of receiving test results via text. Two-way messaging, which has been shown to be more effective in various behavior change interventions compared to one-way interventions [30, 32], could mitigate these challenges and allow women to actively engage in cervical cancer education and services, especially those in resource-limited settings. One study based in Portugal found that adding more than one communication method was more effective than sending only written invitation letters in increasing cervical cancer screening uptake [33, 34]. Their study included a 3-step invitation to screening, in which an automated reminder via text or phone call (step 1), manual phone call (step 2), and face-to-face interview (step 3) were applied sequentially and demonstrated that screening uptake was increased by 17% among women who received the invitation through step 3 compared to those receiving the standard invitation letter. The similar multistep, multimodal system that integrates HPV test result notification via text, phone call, and home visit could be applied in western Kenya to optimize linkage to care.

Our study had several limitations. First, we asked whether enhanced text messaging helped women understand why they needed treatment or how they could access treatment. We relied on self-reporting and did not require participants to share what their understanding was (they simply indicated “yes” or “no”). Second, we only included survey items about the effect of text notifications on the decision-making process with the use of enhanced text notification, and not the use of standard text notification. Therefore, we were not able to accurately compare the varying effects of standard text and enhanced text messaging on treatment acquisition. Third, the measurement of results notification timing for text messages may not be completely accurate, as receipt of the test results was recorded after a message was sent and registered in an active phone; the actual reading of the message was not confirmed by the women. This part of the data collection relied on the transmission of text messages through the Frontline SMS program, in which we did not require a confirmation text to avoid data costs for the women. Fourth, our study did not explore the acceptability of the content in enhanced text messages for participants who sought treatment and those who did not. In contrast, a similar study also developed text messages for women during focus groups but validated the content through interviews with health providers and women, considering both perspectives [35]. Their findings emphasized personalized and persuasive language with a professional tone to encourage women to visit the clinic for their HPV test results. In our study, although we assessed the impact of enhanced text messaging on treatment uptake, we did not directly investigate the effect of the message content or wording on the participants who received these messages. Understanding the impact of specific language and content on women’s decisions to access treatment, their privacy experiences with text-based test results, whether positive or negative, and their perception of sender legitimacy, a crucial element in the context of mobile-based interventions, would have been of immense value. Last, we encountered delays in HPV test kit availability in the middle of the study due to slow customs clearance of the test kits, leading to delays in planned CHCs. This may have contributed to the low uptake of screening and treatment among women. It is also an example of one of the external logistical barriers faced by women in this rural area for which the study could not control.

## Conclusion

In this cohort of women undergoing community-based HPV testing, over three quarters of the participants preferred a cell phone-based strategy (phone call or text messaging) for results delivery. There was no difference in treatment uptake rates between standard and enhanced text groups, even after the text messaging strategy was enhanced with increased messages and adapted content. This enhanced text strategy is one attempt to address low linkage to care in cervical cancer amidst the overall poor transportation, education, and supply resources in Kenya. While enhanced text messaging did not garner higher treatment uptake, reflecting the multiple factors impacting ability to complete the care cascade in in Kenya, it did not result in lower treatment rates or a negative experience for women. As cell phone ownership increases, these results may help programs to provide different options for results notification, though there remains a need to address the structural and logistical barriers that may inhibit women’s decision or ability to follow up with treatment. Future programs could therefore offer multiple results notification methods, including a combination of cell phone-based strategy and home visit, to ensure that they meet the needs of their populations.

## Data Availability

Per the Kenya Medical Research Institute (KEMRI) guidelines, the data will be made available upon reasonable requests to the corresponding author.
